# Stent implantation into the tracheo-bronchial system in rabbits: histopathologic sequelae in bare metal vs. drug-eluting stents

**DOI:** 10.1186/s40348-015-0021-7

**Published:** 2015-11-05

**Authors:** Matthias Sigler, Julia Klötzer, Thomas Quentin, Thomas Paul, Oliver Möller

**Affiliations:** Department of Pediatric Cardiology and Intensive Care Medicine, University Medical Center, Georg August University Göttingen, Robert Koch Strasse 40, D 37075 Göttingen, Germany

**Keywords:** Airway stent, Drug-eluting stent, Tracheomalacia, Granulation tissue, Histology

## Abstract

**Background:**

Stent implantation into the tracheo-bronchial system may be life-saving in selected pediatric patients with otherwise intractable stenosis of the upper airways. Following implantation, significant tissue proliferation may occur, requiring re-interventions. We sought to evaluate the effect of immunosuppressive coating of the stents on the extent of tissue proliferation in an animal model.

**Methods:**

Bare metal and sirolimus-coated stents (Bx Sonic and Cypher Select, Johnson & Johnson, Cordis) were implanted into non-stenotic lower airways of New Zealand white rabbits (weight 3.1 to 4.8 kg). Three stents with sirolimus coating and six bare metal stents could be analyzed by means of histology and immunohistochemistry 12 months after implantation.

**Results:**

On a macroscopic evaluation, all stents were partially covered with a considerable amount of whitish tissue. Histologically, these proliferations contained fiber-rich connective tissue and some fibromuscular cells without significant differences between both stent types. The superficial tissue layer was formed by typical respiratory epithelium and polygonal cells. Abundant lymphocyte infiltrations and moderate granulocyte infiltrations were found in both groups correspondingly, whereas foreign-body reaction was more pronounced around sirolimus-eluting stents.

**Conclusions:**

After stent implantation in the tracheo-bronchial system of rabbits, we found tissue reactions comparable to those seen after stent implantation into the vascular system. There was no difference between coated and uncoated stents with regard to quality and quantity of tissue proliferation. We found, however, a significantly different inflammatory reaction with a more pronounced foreign-body reaction in sirolimus-coated stents. In our small series, drug-eluting stents did not exhibit any benefit over bare metal stents in an experimental setting.

## Background

Stenosis of the tracheo-bronchial system in children may be life-threatening. Reasons include obstructing internal structures (e.g., granulations or tumor), external compression (e.g., lymph nodes or atypical vascular structures), or functional-dynamic changes of the airway wall resulting in tracheomalacia [1, 2]. Preservation of patency of the airways is the aim of any therapeutic intervention [3]. Functional improvement may be achieved by non-invasive ventilation using continuous positive airway pressure which is not tolerated by all patients. It may sometimes not even be sufficient to guarantee adequate ventilation. Accordingly, facing limited alternative therapeutic options, stent implantation into the tracheo-bronchial system may be inevitable and life-saving in otherwise intractable patients [4].

In the beginning, airway stents were made of silicone for relief of subglottic stenosis [5]. Due to the closed design, these stents showed a high rate of migration and could not be adapted to increased size of the airway lumen in a growing individual. These limitations could in part be overcome with the introduction of bare metal stents [6, 7]. In clinical practice, implantation of these stents is an established therapeutic option with proven efficacy and safety [8–10]. The main problem after bare metal airway stents is the formation of obstructing granulation tissue adjacent to the implant [4, 11].

The aim of our study was to evaluate the extent of tissue proliferations after implantation of drug-eluting stents with an anti-proliferative coating. To the best of our knowledge, we report on the use of drug-eluting stents in the trachea-bronchial system in an experimental setting with the longest follow-up period (i.e., 12 months).

## Methods

A total of 28 stents were implanted in the trachea-bronchial system of New Zealand white rabbits (weight 3.1 to 4.8 kg at the time of implantation). Fourteen stents were bare metal coronary stents (Bx Sonic, Johnson & Johnson, Cordis, USA), and 14 had an anti-proliferative coating with sirolimus (Cypher Select, Johnson & Johnson, Cordis, USA), respectively. Re-evaluation and re-dilation (if necessary) of the stents was performed after 1, 3, and 6 months. Finally, the animals were sacrificed after 12 months.

For histopathologic evaluation, tissue blocks containing the implant were dissected with a minimum of surrounding tissue immediately after explantation. After brief flushing with saline, specimens were fixed in formalin (buffered 4 %). Prior to embedding, macroscopic evaluation and photo documentation were accomplished. After fixation, tissue blocks containing the devices were embedded in resin methylmethacrylate (Technovit 9100, Kulzer & Co, Wehrheim, Germany). Following hardening, resin blocks were subsequently sectioned in slices of 0.8 mm using a diamond band saw (300 CP, Exakt GmbH, Norderstedt, Germany). These slices were ground down to 5–30 μm with a horizontal rotatory grinder and polisher (400 CS, Exakt GmbH, Norderstedt, Germany) [12].

Richardson blue was used as a standard histological staining. In order to obtain immunostaining of resin embedded specimen, sections were mounted on glass slides using silicon glue, and deplastification was performed as described previously by our group [13]. Counterstaining of immunohistochemical stains was accomplished with hemalaun. Negative controls were processed without the antigen-specific antibody.

For semi-quantitative grading of histological findings, a 3-grade scoring system was adopted from Schwartz et al. for the following aspects: (a) extent of tissue proliferation within the stent lumen and (b) inflammatory reactions [14].

## Results

A first series of 18 stents (9 Bx Sonic and 9 Cypher Select) was implanted without complications. At the first follow-up study 1 month after implantation, 13/18 stents had been expectorated by the animals (6 Bx Sonic and 7 Cypher Select). The remaining five stents of this group were lost until the next follow-up at 6 months.

A second series of implantations was initiated. Another ten stents were implanted (6 Bx Sonic and 4 Cypher Select). The implantation procedure was modified by implanting the stents with a larger diameter of the balloon in relation to the airway diameter at the site of implantation as documented by preceding bronchography. Only one further stent (Cypher Select) was expectorated until the first follow-up at 1 month. No further stents were lost during the follow-up at 6 and 12 months. Thus, nine stents were available for tissue analysis at the end of the study 12 months after implantation (6 Bx Sonic and 3 Cypher Select). Each of the stents had required re-dilation once during follow-up (Table 1).

**Table 1:** Experimental animals and histology results

Number	Stent type	Weight at implant	Number of re-dilatations	Time of re-dilatation	Histology results			
		Grams			Proliferation^a^	Granulocytes^b^	Lymphocytes^b^	Histocytes/FBR^b^
1	Bx Sonic	4.300	1	1 month	+	++	++	+
2	Bx Sonic	4.170	1	6 months	+	++	++	+
3	Bx Sonic	3.900	1	1 month	+	++	++	+
4	Bx Sonic	4.000	1	1 month	++	+	++	+
5	Bx Sonic	4.300	1	1 month	+	++	++	+
6	Bx Sonic	4.700	1	1 month	+	++	++	+
7	Cypher Select	4.760	1	1 month	+	++	++	+++
8	Cypher Select	4.310	1	6 months	+	+	+	++
9	Cypher Select	4.110	1	1 month	+	++	++	+++

*FBR* foreign-body reaction

^a^Grading of the extent of granulation tissue with the lumen of the stents: 0 – no proliferation; + − lumen obstruction 1–25 %; ++ − lumen obstruction 26–50 %; +++ − lumen obstruction >50 %

^b^Grading of inflammatory reactions describing the presence of different cell types around the stent struts: 0 – no inflammatory cells; + − light, noncircumferential infiltrate; ++ − localized, moderate to dense cellular aggregate; +++ − circumferential, dense cell infiltration

### Macroscopic evaluation

On gross examination, mild to moderate proliferation of whitish material with a smooth glossy surface was seen within the lumen of all stents. There were no signs of local hemorrhage or stent fractures.

### Granulation tissue

**Fig. 1:**
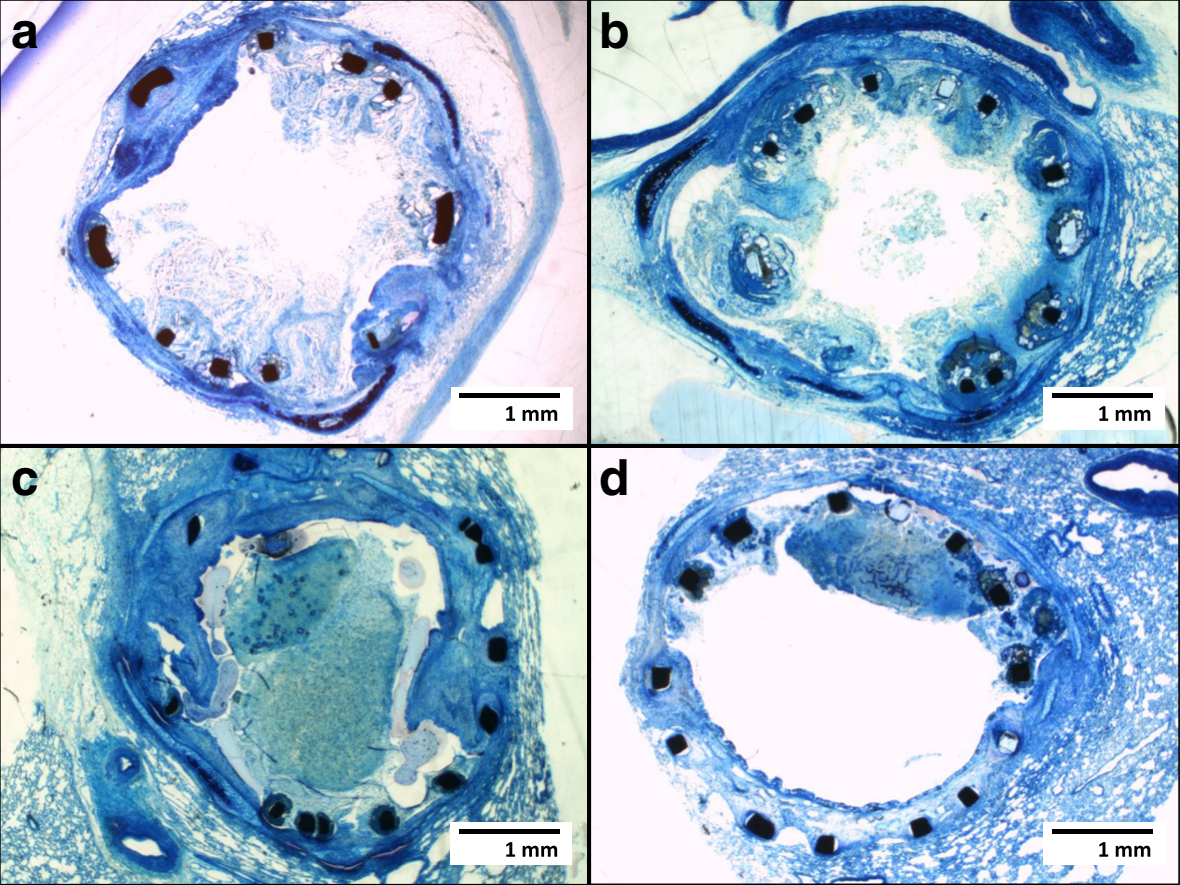
Ground sections (median, Richardson staining) of bare metal stents (**a, b**) and sirolimus-eluting stents (**c, d**) showing tissue proliferations around the stent struts (*black*) in a similar degree; mucus is obstructing the lumen of the stent in **c**

**Fig. 2:**
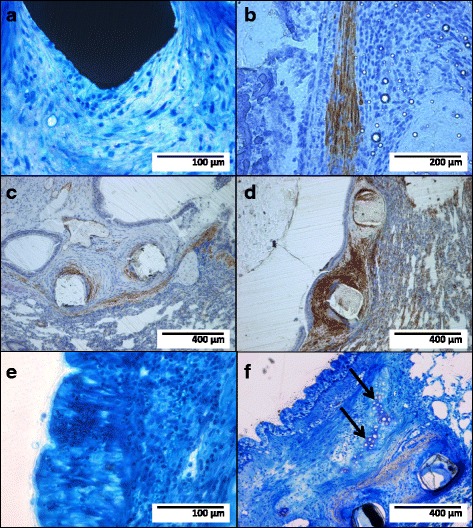
Stent strut (*black structure*, **a**) surrounded by mostly spindle-shaped cells embedded in fiber-rich connective tissue (bare metal stent; Richardson blue staining); immunohistochemical staining (*brown* coloring) with antibodies against smooth muscle myosin (**b**; bare metal stent), vimentin (**c**; sirolimus-eluting stent), and smooth muscle actin (**d**; bare metal stent) identifying the spindle-shaped cells as fibromuscular cells; newly formed epithelium with polygonal cells lining the tracheo-bronchial lumen (**e**; bare metal stent; Richardson blue staining); cartilage structure within the newly formed tissue (**f**, *arrows*; sirolimus-eluting stent; Richardson blue staining)

Histologically, significant proliferation of tissue was found in all specimen around the stent struts (Fig. 1a–d). The proliferations consisted of spindle-shaped cells with the typical morphologic appearance of fibromuscular cells surrounded by fiber-rich connective tissue (Fig. 2a). On immunostaining, fibromuscular cells showed the typical staining pattern with antibodies against smooth muscle myosin (Fig. 2b), smooth muscle actin (Fig. 2c), and Vimentin, a marker for cells of mesenchymal origin (Fig. 2d). There was no difference in quantity or quality of granulation tissue between the two groups of uncoated vs. sirolimus-coated stents (Table 1).

### Epithelialization

On the luminal surface of the newly formed tissue proliferations, respiratory epithelium with the typical pattern of aligned high-prismatic cells with cilia or polygonal cells without cilia had formed (Fig. 2e). In some of the specimens, cartilage structures had developed within the proliferations (Fig. 2f).

### Inflammatory reactions

**Fig. 3:**
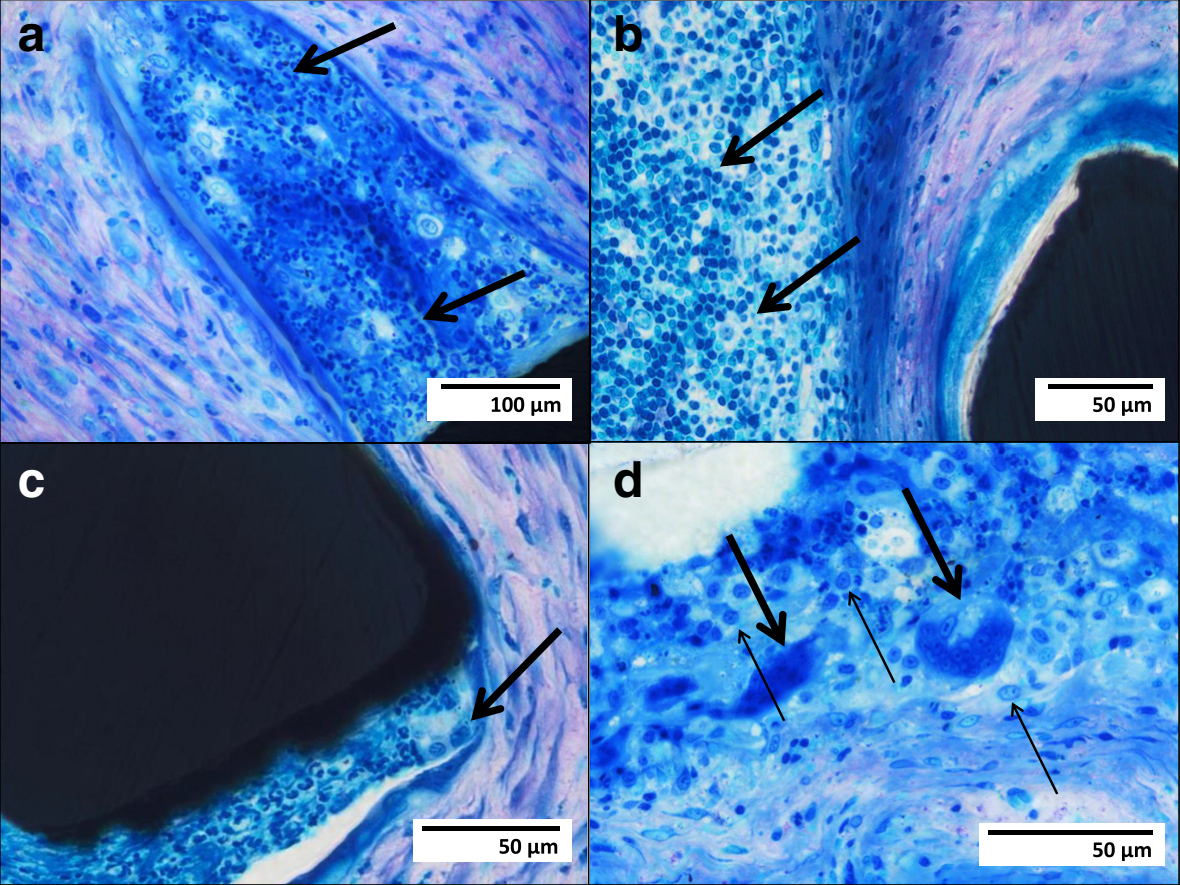
Richardson blue staining of stent struts (*black structures*) with local inflammatory reactions: granulocyte infiltration (*arrows*) locally related to a bare metal stent (**a**); lymphocytic infiltration (*arrows*) locally related to a sirolimus-eluting stent (**b**); only a few histiocytes/macrophages (*arrow*) locally related to a bare metal stent (**c**); multiple macrophages/histiocytes (*light arrows*) and foreign-body giant cells (*bold arrows*) locally related to a sirolimus-eluting stent (**d**)

Dense infiltrations of inflammatory cells were found in all specimens (Table 1). Granulocytes were located predominantly around the stent struts (Fig. 3a), infiltrating the respiratory epithelium (Fig. 2e). Lymphocyte infiltrations showed an irregular distribution pattern through all layers of the airway wall (Fig. 3b). Histiocytes (macrophages) were present in all specimens as well (Fig. 3c), but with a significant accumulation locally related to stent struts of sirolimus-eluting stents. Formation of multiple foreign-body giant cells was also found more pronounced in these stents correspondingly (Fig. 3d).

## Discussion

After stent implantation into the tracheo-bronchial system, formation of granulation tissue with subsequent restenosis is considered the most clinically relevant complication beside stent dislocation [8–10, 15]. It is well-described that inflammatory processes lead to the formation of these tissue proliferations [16, 17].

The main focus of the present study is the reduction of the degree of granulation tissue formation by employing stents with anti-inflammatory coating. This concept was adopted from the field of cardiology. In 2002, a large randomized, double-blind study of bare metal vs. sirolimus-eluting stents in patients with coronary artery disease had shown a significant reduction of late lumen loss in the coated stent group [18]. These results lead to the speculation of improved efficacy of anti-inflammatory coating in stents implanted into the tracheo-bronchial system since the level of inflammatory activity is generally higher in the airways compared to the vascular system. This expectation was further supported by the observation of reduced granulation tissue formation during oral immunosuppressant therapy in 30 adult patients with bare metal airway stents [19].

In our study, however, we could not detect a positive effect of sirolimus coating with regard to granulation tissue formation. All three specimens containing sirolimus-eluting stents showed significantly more foreign-body reaction with multiple macrophages and foreign-body giant cells around the stent struts. There is a well-described relation of foreign-body reaction and formation of granulation tissue [17, 20]. As a consequence, we speculate that the extent of granulation tissue formation may even be more pronounced in sirolimus-coated stents implanted into the tracheo-bronchial system with an implantation time of >12 months.

As in our study, increased inflammatory reactions to drug-eluting stents (compared to bare metal stents) with mainly macrophages but also with lymphocytes were observed in a human coronary atherectomy specimen [21, 22]. Degradable polymers of drug-eluting stents were identified as the main trigger for these reactions [23, 24].

To the best of our knowledge, no implantation of a drug-eluting tracheo-bronchial stent has been reported in humans. Only a few experimental studies examining tracheo-bronchial stents with anti-proliferative coating have been published as of yet. Chao et al. implanted cisplatin-eluting biodegradable stents using the same animal model as in our study. Re-epithelialization and significant inflammatory infiltrates were observed after a follow-up of up to 5 weeks [25]. The extent of granulation tissue formation was not described, and the quality of inflammatory reactions was not further specified. Zhu et al. developed another bioabsorbable tracheal stent with mitomycin C (MMC) drug elution [26]. After a maximum implantation time of 12 weeks in rabbits, the group of animals with drug-eluting stents showed less tracheal obstruction and mucus trapping compared to the bare metal stent group. The latter is the only study to date that directly compares drug-eluting and bare metal stents as it was conducted only by us. We can only speculate that the more favorable results of the study of Zhu et al. might be due to the differing mechanism of the anti-proliferative coating.

### Limitations

Significance of our results is limited due to the small sample size which was related to the high rate of expectorated stents within the first months after stent placement at the beginning of the experiments. Obviously, an undersized balloon diameter chosen for implantation of the stents in non-stenotic airways was the cause for this issue. Expectorations occurred less frequent after modification of the implantation procedure by employing a larger balloon size in relation to the airway diameter at the site of implantation. Furthermore, results were obtained in a rabbit animal model. No systematic data exist on transferability of results to human tissue reactions.

## Conclusions

Our study demonstrates a similar extent of granulation tissue formation in sirolimus-eluting tracheo-bronchial stents in a small experimental series when compared to bare metal stents. Foreign-body reaction was more pronounced in the drug-eluting stent group after an observation period of 12 months which was by far longer than reported in experimental studies in this field before.

### Future perspective

Future studies may focus on new ways to effectively reduce the formation of granulation tissue. This may be achieved by a combination of the principles of drug elution and bioabsorption of the stents in order to avoid chronic inflammation and subsequent granulation tissue formation [26, 27]. Another promising approach may be the implantation of bioresorbable airway splints individually created in a three-dimensional printer which are surgically implanted by external fixation at the region of tracheomalacia as reported by Zopf et al. [28].
